# High Purity Single Wall Carbon Nanotube by Oxygen-Containing Functional Group of Ferrocene-Derived Catalyst Precursor by Floating Catalyst Chemical Vapor Deposition

**DOI:** 10.3390/nano12050863

**Published:** 2022-03-04

**Authors:** Sook Young Moon, Seung-Yeol Jeon, Sung-Hyun Lee, Anna Lee, Seung Min Kim

**Affiliations:** 1 Institute of Advanced Composite Materials, Korea Institute of Science and Technology (KIST), Chudong-ro 92, Bongdong-eup, Wanju-gun 55324, Jeonbuk, Korea; syjeon@kist.re.kr (S.-Y.J.); sunghyun0409@naver.com (S.-H.L.); seungmin.kim@kist.re.kr (S.M.K.); 2 Department of Chemistry, Jeonbuk National University, 567 Baekje-daero, Deokjin-gu, Jeonju-si 54896, Jeonbuk, Korea; annalee@jbnu.ac.kr

**Keywords:** carbon nanotube, direct spinning, floating catalyst chemical vapor deposition, oxygen containing precursor, DFT

## Abstract

Single wall carbon nanotubes (SWCNTs) were synthesized using oxygen-containing ferrocene derived catalysts. The mechanism of synthesizing carbon nanotubes was clarified by the catalyst’s exothermic or endothermic decomposition processes. By monitoring the decomposition process of ferrocene-derived catalyst precursors with and without sulfur, we found that the types of oxygen function groups closely influence catalyst formation and nanotube growth. The ferrocene-derived catalyst precursors have a different oxygen containing groups, which are hydroxyl (–OH, ferrocenenemethanol) and carbonyl (C=O, acetylferrocene, and 1,1′-diacetylferrocene). The sulfur chemical state (S 2p) on synthesized catalyst particles using acetylferrocene and 1,1′-diacetylferrocene has more sulfate (${\mathrm{SO}}_{4}^{2-}$) than others, and there also is a carbon state (C-S-C). The catalyst particle using ferrocenemethanol predominant formed metal–sulfur bonds (such as S^2−^ and $S_{n}^{2-}$). The hydroxyl group (–OH) of ferrocenemethanol enhanced the etching effect to remove amorphous carbon and prevented oxidation on the catalyst particle surfaces; however, the carbonyl group (C=O) of acetylferrocene reacted with the catalyst particles to cause partial oxidation and carbon dissociation on the surface of the catalyst particles. The partial oxidation and carbon contamination on catalyst particles controlled the activity of the catalyst. The DFT study revealed that the ferrocene-derived catalyst precursor was dissociated according to following process: the functional groups (such as CH_3_CO and COH) => first Cp ligands => second Cp ligands. The pyrolysis and release of Fe ions were delayed by the functional groups of ferrocene-derived precursors compared to ferrocene. The thermal-decomposition temperature of the catalyst precursor was high, the decomposition time was be delayed, affecting the formation of catalyst particles and thus making smaller catalyst particles. The size and composition of catalyst particles not only affect the nucleation of CNTs, but also affect physical properties. Therefore, the I_G_/I_D_ ratio of the CNTs changed from 74 to 18 for acetylferrocene and ferrocene, respectively. The purity also increased from 79 to 90% using ferrocene-derived precursors.

## 1. Introduction

Carbon nanotubes (CNTs) have been the subject of research attention in recent years because of the potential applications of their extremely impressive mechanical, electrical, and thermal properties [1,2,3,4,5,6,7]. However, the nanoscale size of CNTs has limited their commercial application to bulk structures. CNT fibers made by the direct spinning method are macroscopic structures that retain their intrinsic properties. Direct spinning by the floating catalyst chemical vapor deposition (FC-CVD) method has been investigated in both academia and industry, owing to its potential for scaled-up production. The FC-CVD process is a very rapid method in which catalyst formation, CNT nucleation and growth, and aerogel formation occur within a few seconds. Therefore, it is difficult to control the process of early stages such as catalyst formation, CNT nucleation, and CNT growth, which are closely linked to the final CNT fiber properties. The individual CNTs constituting CNT fiber tend to be affected by growth conditions, including temperature, flow rate, carrier gas, carbon source, and catalyst composition.

Among many other parameters, catalyst nanoparticles are directly involved in the nucleation and growth of carbon nanotubes. The initial size and mobility of the catalyst significantly affect the CNTs’ formation and configuration. Most previous studies on CNT-fiber synthesis used ferrocene as a catalyst and thiophene as promoter [8,9,10,11]. Because sulfur forms a stable bond with iron according to its low surface energy (FeS, −84.17 kJ/mol), which is lower than α-Fe (−0.19 kJ/mol) [11]. Thus, the iron-catalyst particles demobilize atoms and preserve the coalescence by sulfur atoms during the process. This sulfur contaminates the catalyst particles, leading to deterioration of the catalytic activity [12]. Decreasing catalytic activity in turn causes a decrease in the growth rate, resulting in low-quality CNTs. On the other hand, in previous studies [13,14], water molecules enhanced CNT growth by enhancing and maintaining the activity and lifetime of catalyst particles. This allows the synthesis of defect-free, densely packed and aligned CNTs. Therefore, to enhance catalytic activity, we propose ferrocene-derived precursors containing oxygen groups, which are considered to provide structural control and to enhance catalytic activity. In addition, by controlling the ligand structures, we anticipate that we can control the thermal-decomposition temperatures of catalyst precursors. In FC-CVD, catalyst precursors and promoter thermally decompose at a certain temperature, respectively; then, gaseous atoms form catalyst particles. Ferrocene sublimes at 175 °C. Leonhardt et al., reported the gaseous ferrocene decomposes spontaneously above 497 °C to form metallic ion, indicating that solid or liquid-like Fe particles and different kinds of species exist in the pyrolysis [15,16]. On the other hand, thiophene begins to decompose above 800 °C. The significant difference in decomposition temperature between these materials affects the formation of catalyst particles [11]. Therefore, by delaying thermal decomposition, we can control the size distributions and crystal structures of the catalyst particles at which CNTs start to nucleate and grow. The size distributions dictate the diameter distributions of the synthesized CNTs and also significantly affect their initial growth rates. Additionally, the crystal structure is thought to affect the length of CNTs by affecting the activity and lifetime of the catalyst. Therefore, thermodynamic analysis of the catalyst formation is important and necessary to understand the mechanism of CNT growth. Mohlala et al. reported the synthesis of MWCNT by substituted ferrocene derived catalyst. They revealed that the ferrocene ring substituents influenced both the CNT diameter and the carbon product formed. However, they have not studied catalyst decomposition dynamic effect on growth of CNTs [17].

This study investigates changes in the physical properties and growth mechanism of CNTs according to different functional groups in catalyst precursors. The mechanism of CNT growth was established by analyzing the thermodynamic behavior of catalyst precursors. To the best of our knowledge, this work represents the first report on CNT-fiber growth using a ferrocene-derived catalyst system.

## 2. Materials and Methods

Ferrocene (FeCp_2_, Fe(C_5_H_5_)_2_, 98%, Sigma-Aldrich, St. Louis, MO, USA), acetylferrocene (C_12_H_12_FeO, 95%, Sigma-Aldrich, St. Louis, MO, USA), ferrocenemethanol (C_11_H_12_FeO, 97%, Sigma-Aldrich, St. Louis, MO, USA), and 1,1′-diacetylferrocene (C_14_H_14_FeO_2_, 97%, Sigma-Aldrich, St. Louis, MO, USA) were used without purification as catalysts, just as they were received. Aerogel-like carbon nanotubes were synthesized via the floating catalyst chemical vapor deposition (FC-CVD) method (Figure S1). The stock solution comprised acetone, organometallic compounds, and thiophene (C_4_H_4_S, ≥99%, Sigma-Aldrich, St. Louis, MO, USA); this solution was injected into a reactor at a rate of 12 mL/h and a heating zone temperature of 1200 °C. The ferrocene derived organometallic compound-to-thiophene molar ratio was set to 1 (mol/mol). Hydrogen was used as the carrier gas. The aerogel-like carbon nanotubes were synthesized in the outstream area and collected in the final chamber.

The synthesized CNTs were characterized through Raman spectroscopy (Renishaw, in a via excited by a 514-nm laser), transmission electron microscopy (TEM, Technai G2 F20, FEI, Hillsboro, OR, USA), field-emission scanning electron microscopy (FE-SEM, Verios 460, FEI, Hillsboro, OR, USA), X-ray photoelectron spectroscopy (XPS, K-Alpa, Thermo Fisher Scientific, Walthan, MA, USA), and X-ray diffraction (XRD, by Cu Kα radiation, 45 kV, 200 mA, Smartlab, Rigaku, Tokyo, Japan). The thermodynamic reaction of catalyst precursors was analyzed through thermogravimetric-differential scanning calorimetry (TG-DSC, Labsys Evo, Setaram, Caluire, France). The heating rate was 10 °C/min with a low of Ar. The decomposed gas analyzed by process gas monitor with mass spectroscopy (BGM202, Ulvac, Chigasaki, Japan)

Density functional theory (DFT) simulation were carried out using the plain-wave QUANTUM ESPRESSO package [18]. Generalized gradient approximation (GGA) was used with the Perdew–Burke–Ernzerhof (PBE) functional of ultra-soft pseudopotentials. The kinetic energy cutoff was set as 80 Ry with convergence criteria of 10^−2^ eVÅ^−1^. Total energy of isolated molecule was calculated at the gamma point in a cubic cell with a 30 Å edge length. All molecules of ferrocene and ferrocene derivatives were fully relaxed before self-consistent field (SCF) calculation. The bond dissociation energy was obtained by comparing the difference in energy before and after removing atoms/fragments from the molecule.

## 3. Results

**Figure 1:**
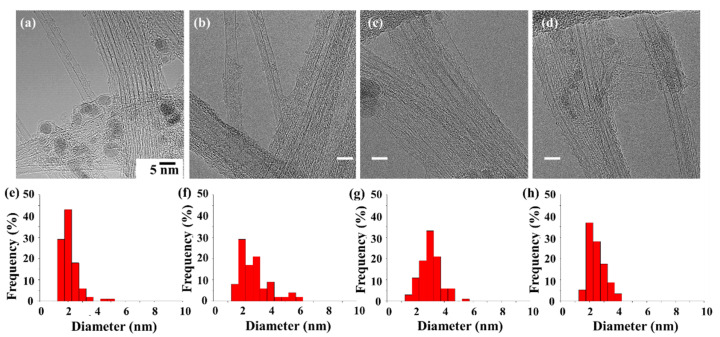
TEM images (**a**–**d**) and size distribution (**e**–**h**) of synthesized CNTs with different catalyst precursors: (**a**) ferrocene, (**b**) acetylferrocene, (**c**) ferrocenemethanol, and (**d**) 1,1′-diacetylferrocene.

In this study, large-scale, high-quality single-walled CNT (SWCNT) and a few-walled CNT (FWCNT) with a narrow-diameter distribution were synthesized by FC-CVD. All synthesis conditions were the same except for types of catalyst precursors. The synthesized CNTs almost showed SWCNTs, but the acetylferrocene-based product shows SWCNTs and a few FWCNTs (Figure 1). The CNT diameters exhibited their narrowest distribution with ferrocene. After sufficient dispersion by sonication, 100 CNTs were selected for each sample and the diameter distribution was analyzed. When the CNT was synthesized with ferrocene, the tube diameter (d) ranged from 1.1 to 4.8 nm; however, most of the d existed at 1~2.5 nm. When the CNT was synthesized with acetylferrocene, the distribution range of d was increased and measured between 1.5 and 6 nm, with most falling in the range of 1.5~3 nm. The synthesized CNT shows a mixed structure with SWCNT and FWCNTs. Meanwhile, when CNT was synthesized with ferrocenemethanol, the size distribution narrowed and d decreased to between 1.5 and 5.1 nm, with a large portion being in the range of 2.5~3.5 nm. The average diameter was larger than that using ferrocene. When the CNT was synthesized with 1,1′-diacetylferrocene, the diameter was found to be between 1.5 and 3.6 nm, and a substantial portion fell in the range of 2~3 nm. The tube diameter decreased less than for other ferrocene-derived catalysts. 

As a result of classifying the types of synthesized CNTs by precursor, the synthesized CNTs are mostly SWCNTs, but other types of CNTs such as FWCNTs and MWCNTs also exist (Figure S2). For acetylferrocene, the proportion of FWCNTs is increased compared to other precursors. This confirms the possibility of synthesizing other types of CNTs such as DWCNTs. In addition, in the case of ferrocenemethanol, many structures were found in which the double wall of the intermediate formation process was piled up on the outer wall of the SWCNT. Therefore, it was included as SWCNT, but there is ambiguity in the classification process. In the case of 1,1′-diacetylferrocene, the yield of the tube itself was significantly lower than that of other precursors. In the case of 1,1′-diacetylferrocene, the yield of the tube itself was significantly lower than that of other precursors, but the synthesized CNTs were mostly SWCNTs. In addition, there were many tube-shaped carbon laminates, but they were excluded from the number of tubes.

**Figure 2:**
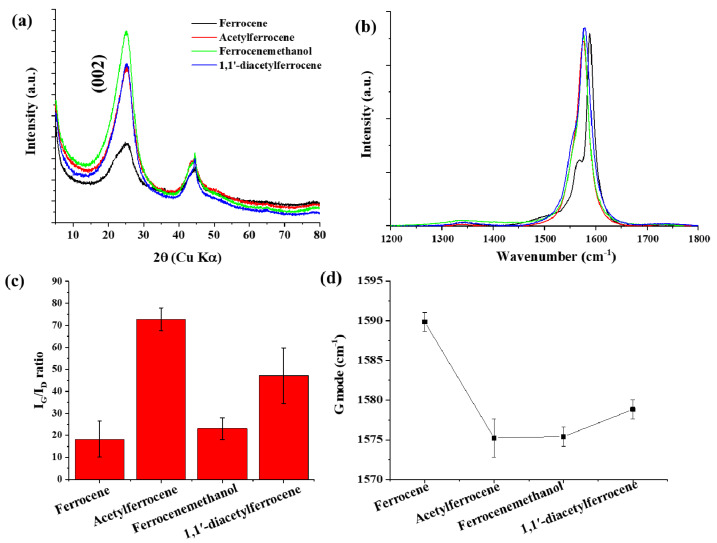
(**a**) X-ray diffraction patterns, (**b**) Raman spectra of CNTs synthesized with different catalyst precursors, (**c**) I_G_/I_D_ ratio, and (**d**) G mode.

The as-synthesized CNTs showed high crystallinity and all samples showed two main peaks between 1000 cm^−1^ and 2000 cm^−1^, which corresponded to the D-band and the G-band (sp^2^) [19,20,21]. The high I_G_/I_D_ ratio was observed in acetylferrocene (I_G_/I_D_ = 74.43) (Figure 2). The I_G_/I_D_ ratios for ferrocenemethanol and 1,1′-diacetylferrocene were 23.06 and 47.12, respectively, each of which was larger than the value when ferrocene was used (I_G_/I_D_ = 18.31). The different pyrolytic reactions caused by using different catalyst precursors are thought to have produced catalysts more suitable for CNT growth. The best CNT synthesis was observed for acetylferrocene. The G-mode caused by the bond stretching of all pairs of sp^2^ atoms in both rings and chains. The peak shift of the G-mode can be associated with axial elongation/shortening of the C–C bonds in a nanotube shell. In case of SWCNT, the G band shift also related with diameter. The G-mode peaks down shifted by approximately 16 cm^−1^ with catalyst precursors. In addition, C (002) peak on XRD patterns from ferrocene derived samples increased compared to using ferrocene, which means increasing crystallinity.

The radial breathing mode (RBM) of Raman spectra correspond to the atomic vibration of the C atoms in the radial direction. It is used to define the diameter of SWCNTs. The bundle of SWCNT diameters according to the expression [22]:

(1) $$\omega_{\mathrm{RBM}}=\frac{234}{d_{t}}+10\;\;$$

where $\omega_{\mathrm{RBM}}$ is the RBM Raman shift, and *d_t_* is diameter. The RBM spectra of all samples shows in Figure S3. RBM peaks were measured at 20 positions for each sample, and were fitted with Lorentzian. The bundled nanotube fibers have broad RBMs, and it is difficult to trace all the peaks corresponding to each individual CNT that will be present [23]. The broad RBMs have emerged due to the microstructure of heavily bundled nanotubes with varying diameters. In the case of acetylferrocene, the entire peak position shifts to a lower band compared to ferrocene, which means that the diameter of CNTs is larger than that of ferrocene. The diameter was calculated in the range of 1.0–2.25 nm by Equation (1). For ferrocene, the calculated diameter ranged from 0.9–1.6 nm, and for ferrocenemethanol it was in the range from 1.0–2.3 nm. In the case of 1,1′-diacetylferrocene, the diameter was in the range of 1.0–1.7 nm. The difference between the CNT diameter measured using the measurement software (image J) in the TEM image and the value measured using the Raman RBM is thought to be due to the inability to fit the peaks for all individual CNTs in the broad RBM peak as described above [23]. In addition, in the case of carbon nanotubes measured by a TEM image, the average value calculated by being measured in a range of all sizes may increase. However, in the case of RBM, the peaks less than 100 cm^−1^ are not measured, which may cause errors. However, as can be seen from the TEM image, it was confirmed that the diameter of CNTs using the ferrocene-derived precursor was larger than that of ferrocene.

A thermo-gravimetric (TG) analysis is used to indicate the purity and degree of graphitization of CNT structures. The results show that the amounts of residual mass decreased from 21 to 10% depending on the catalyst precursors (Figure S4). Moreover, the thermal-decomposition temperature increased with ferrocene-derived catalysts except for 1,1′-diacetylferrocene. The high-purity CNTs that appear in the cases of acetylferrocene and ferrocenemethanol have the lowest amount of residual mass (10%). The pyrolysis of ferrocenemethanol was higher than that of acetylferrocene because of CNT structures. Liew et al. [24] reported the thermal stability of SWCNT and MWCNTs; they found that SWCNTs are thermally more stable than MWCNTs. Because atoms from different layers start to vibrate at high temperature and atoms from one layer collide with those from the neighboring layers, it is easier for the MWCNTs to be destroyed. In their study, simulations also indicate that the CNTs with larger diameter are also more resistant to thermal loads. Therefore, CNTs synthesized using ferrocene-derived catalyst precursors are more thermally stable than the ferrocene used. Meanwhile, when we used 1,1′-diacetylferrocene, the CNTs showed faster pyrolysis than in other cases because there exist both SWCNTs and tube-like carbon-stacking agglomerate (Figure S5).

**Figure 3:**
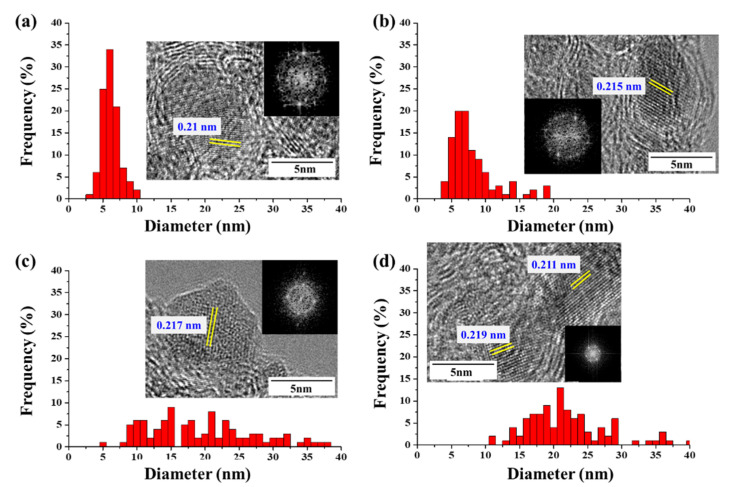
TEM images and size distribution of catalyst particles at CNT bundles with different catalyst precursors: (**a**) ferrocene, (**b**) acetylferrocene, (**c**) ferrocenemethanol, and (**d**) 1,1′-diacetylferrocene.

The diameters and lengths of CNTs are known to be related to the size and structure of the catalyst particles. We measured the catalyst-particle-size distributions in the CNT bundle (Figure 3); when CNTs were synthesized using ferrocene, the catalyst particles had a size distribution between 2 and 10 nm, but most were under 7 nm. When the CNT was synthesized using acetylferrocene, the catalyst particles measured between 3.59 and 19.04 nm and the distribution range was broadened compared to that obtained with ferrocene. However, most particles had diameters between 4 nm and 8 nm with an average size of approximately 7.72 nm similar to that of ferrocene. Meanwhile, when CNTs were synthesized using ferrocenemethanol, the particle size was between 5 and 38 nm with a wide distribution range. The average size increased significantly to 18.97 nm. By contrast, at 1,1′-diacetylferrocene, the particle sizes also increased, and the distribution was broad, falling in the range of 11~40 nm. However, most particle sizes were between 17 nm and 24 nm. If the size of the catalyst particles was too large, they were not expected to grow high-quality SWCNTs. This result explains why FWCNT productivity was increased using acetylferrocene. On the other hand, particles produced with ferrocenemethanol are strongly faceted, with sharp corners, while the particles produced with ferrocene, acetylferrocene, and 1,1′-diacetylferrocene were rounded. Yamada et al. [25] observed the alteration of carbon-coated Fe catalysts into flatter particles upon the removal of the carbon coating by water treatment. The –OH group on ferrocenmethanol reacts like water molecules in water-assisted CVD to aid in the growth of nanotubes and control particle shape. On the other hand, acetylferrocene produced C=O during decompositions. Dee et al. reported the benefits of using controlled exposure to carbon for catalyst reduction by carbothermal reduction. Preloading carbon accelerates catalyst-nanoparticle formation via film dewetting and inhibits Ostwald ripening, thereby increasing the probability of CNT nucleation and the resultant density of the CNT population [26]. Thus, preloaded carbon might extend the lifetime of the catalyst nanoparticles by reduction of iron oxide (Fe_x_O_y_), which does not allow for this pathway to catalyst deactivation. Additionally, the lattice spacing of the catalyst with acetylferrocene showed 0.215 nm, which correspond to γ-Fe. Wirth et al. reported that the additional preloading carbon can be diffused into Fe and formed meta-stable γ-Fe phase [27,28]. However, over preload carbon species enhanced particle formation and graphitic encapsulation. As a result of that 1,1′-diacetylferrocene showed many of graphitic layer capped catalyst particles because of their double function groups. Additionally, the molecular oxygen generation will preferentially react with smaller diameters with larger curvature due to the weakened C-C bonding induced by bond bending [26]. Thus, the SWCNTs with small diameters were removed and the CNT showed a large diameter when we used acetylferrocene and ferrocenemethanol.

**Figure 4:**
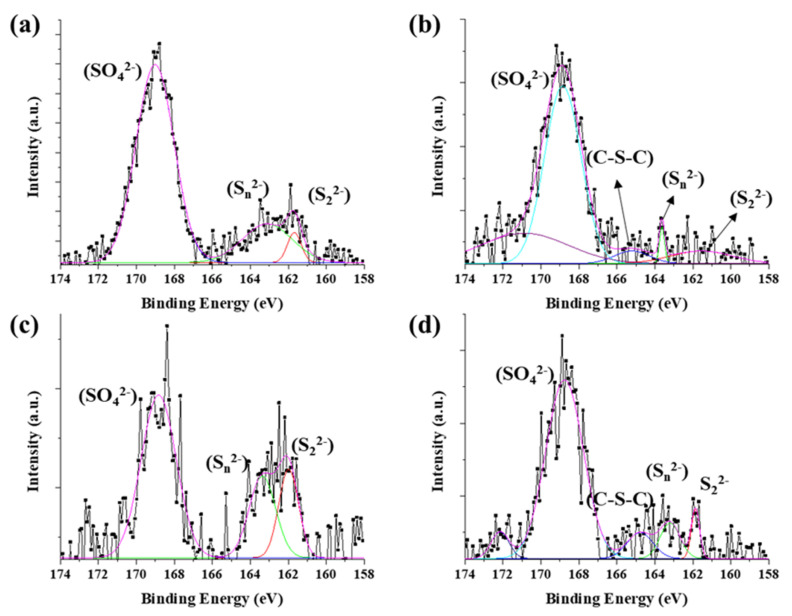
XPS spectra of S 2p on CNT bundle: (**a**) ferrocene, (**b**) acetylferrocene, (**c**) ferrocenemethanol, and (**d**) 1,1′-diacetylferrocene.

The chemical state of synthesized byproduct is also related to catalytic activity. The surface chemical states of the catalyst particles in CNT bundle were identified by X-ray photoelectron spectroscopy (XPS) (Figure 4). The peaks were analyzed by Gaussian peak fitting. The Fe 2p 3/2 and Fe 2p 1/2 orbitals were identified as corresponding to the peaks at 710 eV and 724 eV, respectively (Figure S6). The Fe 2p 3/2 could be divide into three peaks at biding energies of 707.7 eV, 709.8 eV, and 711.1 eV. The peaks assigned to Fe(II)-S (707.1 eV), and Fe(III)-S (709.8 eV, and 711.1 eV). The S 2p shows more complex state with acetylferrocene and 1,1′-diacetylferrocene compared with ferrocene and ferrocenemethanol, which assigned to 161.5 eV (S^2−^), 163.48 eV ($S_{n}^{2-}$), 165.2 eV (C-S-C), and 168.8 eV (${\mathrm{SO}}_{4}^{2-}$) (Figure 4b). The sulfate highly appeared in surface than ferrocene. Only these two precursors have a C-S-C (165.2 eV) bond unlike other precursors. It can be considered that the acetyl group (CH_3_CO) possessed by the precursor had an effect on the formation of the catalyst particles.

However, ferrocenemethanol mainly formed metal-sulfur bonds (such as S^2−^ and $S_{2}^{2^{-}}$) than others. It means the –OH groups react the same as water molecules which prohibit oxidation. Therefore, ferrocenemethanol shows a lower oxidation state than the other catalysts. However, the formation of the predominant metal-sulfur bond reduced the activity of the catalyst for synthesizing CNTs and reduced the diffusion rate of carbon. In a previous study [29], it was confirmed that the oxidized state of iron sulfide helps CNT growth, but the fully crystallized to FeS_2_ could not role of catalyst for CNT growth. The sulfur-metal peaks increase in the following order: ferrocenemethanol > ferrocene > 1,1′- diacetylferrocene > aetylferrocene. On the other hand, in the case of 1,1′-diacetylferrocene, the catalytic activity was reduced compared to acetylferrocene during catalyst nucleation due to initial carbon contamination under the influence of the acetyl group, which was confirmed by the appearance of many C-S-C bonds.

The catalyst particles were formed by two steps in our experiment; decomposition of the catalyst precursor (Fe) and promoter (S), followed by recrystallization of Fe-S compounds. To understand why the ferrocene-derived catalyst precursor enhanced the properties, nucleation, and growth of CNTs, the catalyst-decomposition process was investigated herein through TG-DSC. However, thiophene is not suitable for TG-DSC experiments because of its high volatility; therefore, sulfur was used as the source of S, rather than thiophene.

**Figure 5:**
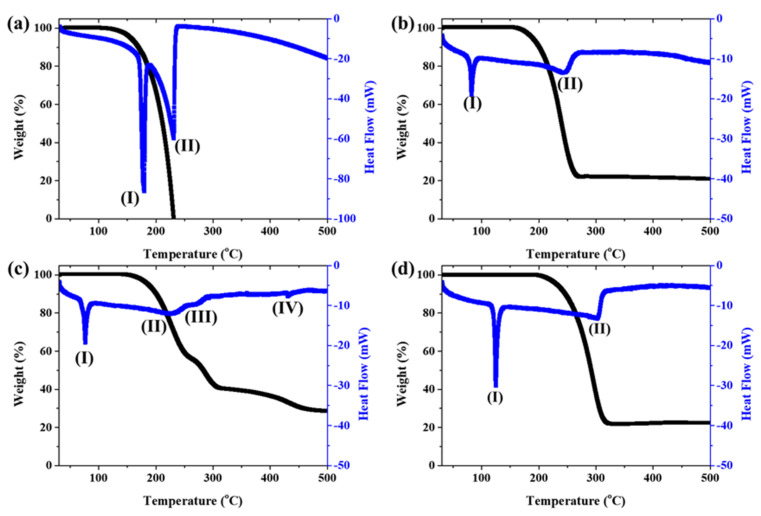
TG-DSC of catalyst precursors: (**a**) ferrocene, **(b**) acetylferrocene, (**c**) ferrocenemethanol, and (**d**) 1,1′-diacetylferrocene.

**Figure 6:**
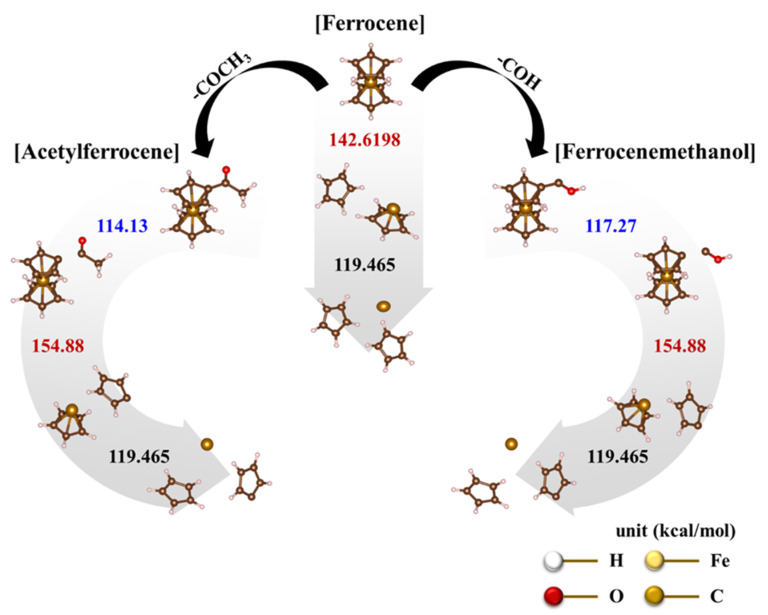
Dissociation mechanisms of catalyst precursors based on thermodynamic bond dissociation energy (BDE): ferrocene, acetylferrocene, and ferrocenemethanol.

At first, only ferrocene and ferrocene-derived precursors without sulfur were analyzed for TG-DSC up to 600 °C (Figure 5). Additionally, bond dissociation energy was calculated by DFT simulations to track the catalyst’s decomposition mechanism (Figure 6). The endothermic reaction appears at 178 °C (Figure 5a(I)) and 231 °C (Figure 5a(II)) in ferrocene. This temperature range is due to the sublimation reaction. Ferrocene decomposes above 497 °C to form a metallic ion, indicating that solid or liquid-like Fe particles and different kinds of species exist in the pyrolysis. However, the amount of sample remaining after sublimation is too small to confirm the next step of pyrolysis, so it cannot be confirmed with TG.

However, the calculated bond-dissociation energies are 142.62 kcal/mol and 119.5 kcal/mol, which correspond to the dissociation of C_p_ ligands in the first and second layers, respectively [30,31]. The first dissociating C_p_ ligand needs to have a higher energy than the second layer. Thus, the ferrocene-dissociation mechanism involves the heterolytic dissociation according to the following steps:

C_p_^−^ + [C_p_Fe]^+^ → C_p_^−^ + C_p_^−^ + Fe^2+^

On the other hand, ferrocene derived catalysts show more complex pyrolysis reactions. The first endothermic reactions in acetylferrocene and ferrocenemethanol occurred at 83 °C (Figure 5b(I)) and 77 °C (Figure 5c(I)), and the second endothermic reactions appeared at 246 °C (Figure 5b(II) and 226 °C (Figure 5c(II)), respectively. Unlike the first endothermic reaction, the second reaction of acetylferrocene occurred later than that of ferrocenemethanol. In addition, ferrocenemethanol undergoes another endothermic reaction at 270 °C (Figure 5c(III)). The ferrocene-derived catalyst precursor firstly dissociated the functional groups such as CH_3_CO and COH from the ferrocene structure. The dissociation-binding energies of acetylferrocene and ferrocenemethanol were 114.14 kcal/mol and 117.27 kcal/mol, and the first-layer dissociation energy after detachment of the functional groups was 154.88 kcal/mol. This suggests that more energy is needed to decompose than ferrocene (142.62 kcal/mol). Thus, the ferrocene-derived precursors decomposed at a higher temperature than did ferrocene. The decomposition process of each precursor with bond dissociation shows Figure 6. We assumed that, if the thermal-decomposition temperature of the catalyst precursor was high, the decomposition time would be delayed, affecting the formation of catalyst particles and thus making smaller catalyst particles. Indeed, the pyrolysis and release of Fe ions were delayed by the functional groups of ferrocene-derived precursors, which were related to the bond-dissociation energy. However, the catalyst-size distribution and average diameter of the ferrocene-derived precursors were broader and larger than those of ferrocene itself. In general, the reactivity of a catalyst decreases as its size increases, resulting in low nucleation and growth. However, in our case, it was possible to synthesize a highly crystalline CNT despite the large catalyst particles. Therefore, we believe that the chemical state and crystalline structure of the catalyst particles plays a more important role in the formation of CNTs because of the addition of sulfur.

**Figure 7:**
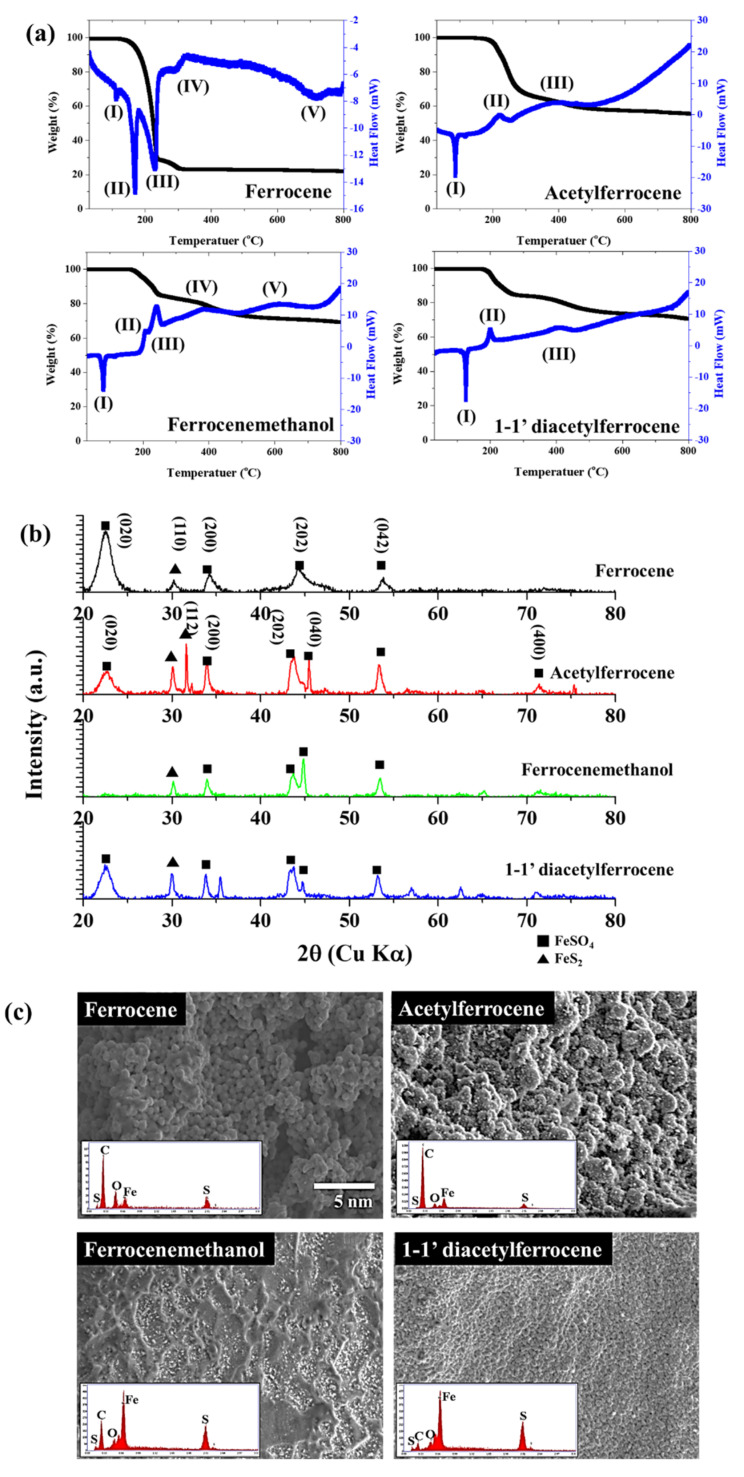
(**a**)TG-DSC about catalyst precursors with sulfur, (**b**) XRD patterns, and (**c**) SEM images and EDS about catalyst precursors with sulfur after thermal decomposition.

To confirm the reactivity with sulfur during pyrolysis, all catalyst precursors were pyrolyzed with sulfur (Fe:S = 1:1 (at %)) up to 800 °C. The states of the formed particles between ferrocene-thiophene and ferrocene-sulfur are different, but the reactivity with sulfur atoms can be estimated. The generation of secondary species differed according to the oxygen containing functional group on the precursors. The crystalline structures were examined by X-ray diffraction (XRD) (Figure 7b), and the catalyst particles in ferrocene-sulfur system were well-assigned to FeSO_4_ (JCPDS No. 12-0068). However, other ferrocene-derived catalysts show mixed structures, which indexed FeSO_4_ (JCPDS No. 12-0068) and FeS_2_ (JCPDS No. 2-0908). The newly formed FeS_2_ nuclei further grow into nanoparticles, and the ferrocene-derived precursors react rapidly with sulfur to form sulfur compounds unlike ferrocene. Thus, the concentration of residual compounds increased compared with ferrocene. Exothermic peaks appear at 222 °C (Figure 7a(II)), 240 °C (Figure 7a(II,III), and 200 °C (Figure 7a(II)), corresponding to acetylferrocene, ferrocenemethanol, and 1,1′-diacetylferrocene, respectively. This exothermic peak means that crystallization and oxidation of the mixed compounds occurs faster than the reaction of ferrocene at 320 °C. Fe^2+^ ions are generated, and sulfur ions and un-reacted sulfur will immediately combine with them to form FeS_2_ [29]. The DSC of elemental sulfur mainly comprises three endothermic signals: the α→β transition of sulfur at around 100 °C, the melting at ~120 °C, and the λ-transition, which starts at ~160 °C. The sulfur was completely sublimated at ~330 °C [32]. The reaction of ferrocene-derived precursors with sulfur occurs as follows: first, the functional group detaches from the precursors; then, melted sulfur reacts with unsaturated ferrocene-derived molecules; finally, sublimation and recrystallization occur. The synthesized catalyst particles aggregate and form a non- spherical morphology in contrast to what happens for ferrocene, because it is driven by the minimization of interfacial energy. Thus, the average diameters of the ferrocene-derived precursors are broader and larger than those formed from ferrocene.

**Figure 8:**
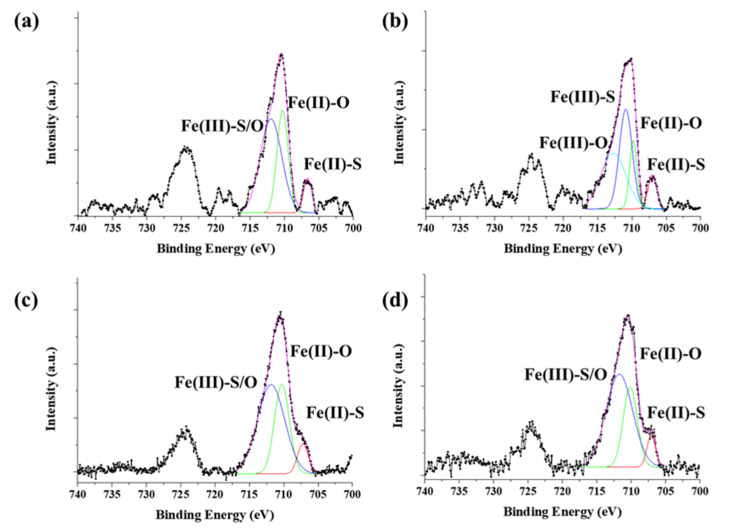
XPS spectra of Fe 2p of thermally decomposed catalyst precursors with sulfur: (**a**) ferrocene, (**b**) acetylferrocene, (**c**) ferrocenemethanol, and (**d**) 1,1′-diacetylferrocene.

The chemical state of synthesized byproduct is also related to catalytic activity. The surface chemical states of the catalyst particles were identified by X-ray photoelectron spectroscopy (XPS) (Figure 8). The Fe 2p 3/2 and Fe 2p ½ orbitals were identified as corresponding to the peaks at 710 eV and 724 eV, respectively. The peaks were analyzed by Gaussian peak fitting and assigned to Fe^2+^ and Fe^3+^. For ferrocene, three peaks at 706.6 eV (Fe(II)-S), 710.3 eV (Fe(II)-O), and 711.9 eV (Fe(III)-S/O) were observed; these were assigned to Fe^2+^. For acetylferrocene, four peaks were observed at 707 eV (Fe(II)-S), 709.8 eV (Fe(II)-O), 710.9 eV (Fe(III)-S), and 712.7 eV (Fe(III)-O), which were assigned to mixtures Fe^2+^ and Fe^3+^. The generation of Fe^3+^ requires an unspecified oxidative process, leaving some ambiguity as to the oxidation state of the iron formed directly from ferrocene-derived precursor dissociation [27]. On the other hand, for ferrocenemethanol, three peaks were observed at 707.2 eV (Fe(II)-S), 710.2 eV (Fe(II)-O), and 711.8 eV (Fe(III)-S/O), for which the chemical state was similar to that of ferrocene. 1,1′-diacetylferrocene also shows results similar to those of ferrocene and ferrocenemethanol, which assigned as 707.11 eV (Fe(II)-S), 710.2 eV (Fe(II)-O), 711.7 eV (Fe(III)-S/O). The S 2p shows more complex state with acetylferrocene and 1,1′-diacetylferrocene compared with ferrocene and ferrocenemethanol, which assigned to 160.92 eV (S^2−^), 162.11 eV ($S_{2}^{2^{-}}$), 163.48 eV ($S_{n}^{2^{-}}$), 164.58 eV (S_0_), and 167.90 eV ($S_{2}O_{3}^{2^{-}}$) (Figure S7). The oxidation form highly appeared in the surface than ferrocene. However, ferrocenemethanol mainly formed metal–sulfur bonds (such as S^2−^ and $S_{2}^{2^{-}}$) than others. It means the –OH groups react as water molecules which prohibit oxidation. Therefore, ferrocenemethanol shows a lower oxidation state than the other catalysts. As we mentioned previously, ferrocene and ferrocene derived precursors produce various substances upon pyrolysis. These substances affect when it combines with the decomposed sulfur to form catalyst particles. In the process of synthesizing CNTs in which a carbon source is actually supplied, functional groups derived from ferrocene help to activate the catalyst, but when only pyrolysis of the catalyst was observed in an inert atmosphere, these functional groups acted as sources to form compounds. However, it was confirmed that the functional groups of the initial catalyst precursor control the reactivity of iron ions and the structure of the catalyst.

## 4. Conclusions

We have demonstrated a method for synthesizing high-quality SWCNTs and FWCNTs with tunable diameters and purities using ferrocene-derived catalyst precursors. The presence of oxygen containing functional groups on catalyst precursors affects CNT physical properties. The hydroxyl group (–OH) in ferrocenemethanol removed carbon contamination and to reduced oxidation on catalyst particles. It resulted in the formation of high metallic Fe-S/S_2_ bond. These sulfide materials reduced catalytic activity and prevented dissociative adsorption of carbon on the catalyst-particle surfaces. The carbonyl group (C=O) of acetylferrocene resulted to partial oxidation and carbon contamination on the catalyst particles. It modulates the reactivity of the catalyst and effects on CNT nucleation and physical properties.

The decomposition behavior of catalyst precursors was confirmed through DFT study. Unlike ferrocene, the ferrocene-derived precursor is decomposed in three steps. First, the functional group is separated, secondly the first C_p_ is cleaved, and finally the remaining C_p_ is cleaved. Due to these thermal decomposition properties, the catalyst particle size of the ferrocene-derived catalyst precursor is reduced because the formation rate of catalyst particles is slower than that of ferrocene. Therefore, the I_G_/I_D_ ratio changed from 18 to 74, and the purity increased from 79 to 90% using ferrocene-derived precursors. However, the excessive carbonyl group (1,1′-diacetylferrocene) rather interfered with the CNT formation because of the formation of a graphitic layer on the catalyst surface. The catalyst particles are polluted too much before CNT nucleation, which significantly reduces the catalyst activity, and eventually the catalyst is covered with a carbon layer, making it impossible to grow CNTs.

## Data Availability

The data presented in this study are available on request form the corresponding author.

## Supplementary Materials

The following supporting information can be downloaded at: https://www.mdpi.com/article/10.3390/nano12050863/s1, Figure S1. FC-CVD system in this experiment and temperature gradient on hot zone: Figure S2. Morphology of CNTs with different catalyst precursors: (a–c) ferrocene, (d–f) acetylferrocene, (g–i) ferrocenemethanol, (j–l) 1,1′-diacetylferrocene, and (m) frequency of tube type: Figure S3. RBM mode of Raman spectra: (a) ferrocene, (b) acetylferrocene, (c) ferrocenemethanol, (d) 1,1′-diacetylferrocene, and (e) calculated tube diameter according to RBM peak and TEM image: Figure S4. TG-DSC of the synthesized CNTs with catalyst precursors: (a) ferrocene, (b) acetylferrocene, (c) ferrocenemethanol, and (d) 1,1′-diacetylferrocene: Figure S5. TEM images of carbon stacking morphology synthesized with 1,1′-diacetylferrocene: Figure S6. XPS spectra of Fe 2p of catalyst particles in CNT bundle: (a) ferrocene, (b) acetylferrocene, (c) ferrocenemethanol, and (d) 1,1′-diacetylferroce: Figure S7. XPS spectra of S 2p of thermally decomposed catalyst precursors with sulfur: (a) ferrocene, (b) acetylferrocene, (c) ferrocenemethanol, and (d) 1,1′-diacetylferroce.
