# Supplementary material for: High Purity Single Wall Carbon Nanotube by Oxygen-Containing Functional Group of Ferrocene-Derived Catalyst Precursor by Floating Catalyst Chemical Vapor Deposition
Source: Nanomaterials (Basel). 2022 Mar 4;12(5):863. doi: 10.3390/nano12050863 (PMC8912279; doi:10.3390/nano12050863)
Supplement: Supplementary file 1 [file nanomaterials-12-00863-s001.zip › nanomaterials-1581026-supplementary.pdf]

nanomaterials

Supplementary

High purity single wall carbon nanotube by oxide-containing functional group of ferrocene-derived catalyst precursor by floating catalyst chemical vapor deposition

Sook Young Moon [1,*], Seung-Yeol Jeon [1], Sung-Hyun Lee [1], Anna Lee [2] and Seung Min Kim [1]

1 Institute of Advanced Composite Materials, Korea Institute of Science and Technology (KIST), Chudong-ro 92, Bongdong-eup, Wanju-gun, Jeonbuk 55324, Korea; syjeon@kist.re.kr (S.-Y.J.) sunghyun0409@naver.com (S.-H.L.); seungmin.kim@kist.re.kr (S.M.K.)
2 Department of Chemistry, Jeonbuk National University, 567 Baekje-daero, Deokjin-gu, Jeonju-si, Jeonbuk 54896, Korea; annalee@jbnu.ac.kr
* Correspondence: moon.sookyoung@kist.re.kr

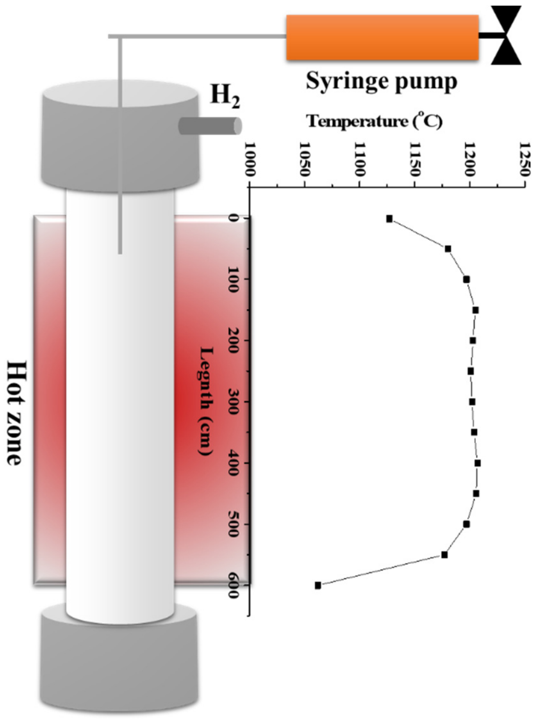

Figure S1. FC-CVD system in this experiment and temperature gradient on hot zone.

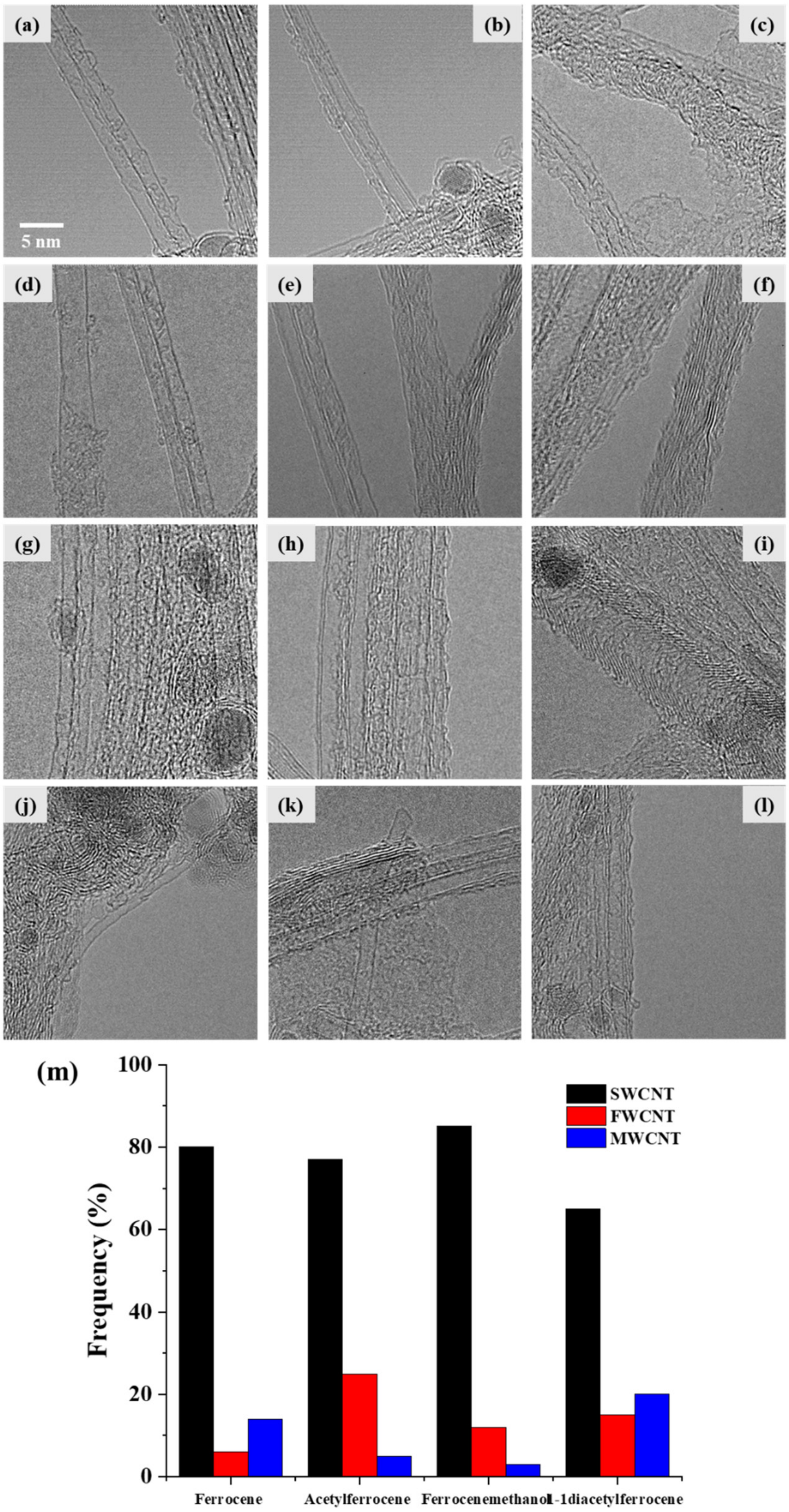

Figure S2. Morphology of CNTs with different catalyst precursors: (a) –(c) Ferrocene, (d)-(f) Acetylferrocene, (g)-(i) Ferrocenemethanol, (j)-(l) 1,1′-diacetylferrocene, and (m) frequency of tube type.

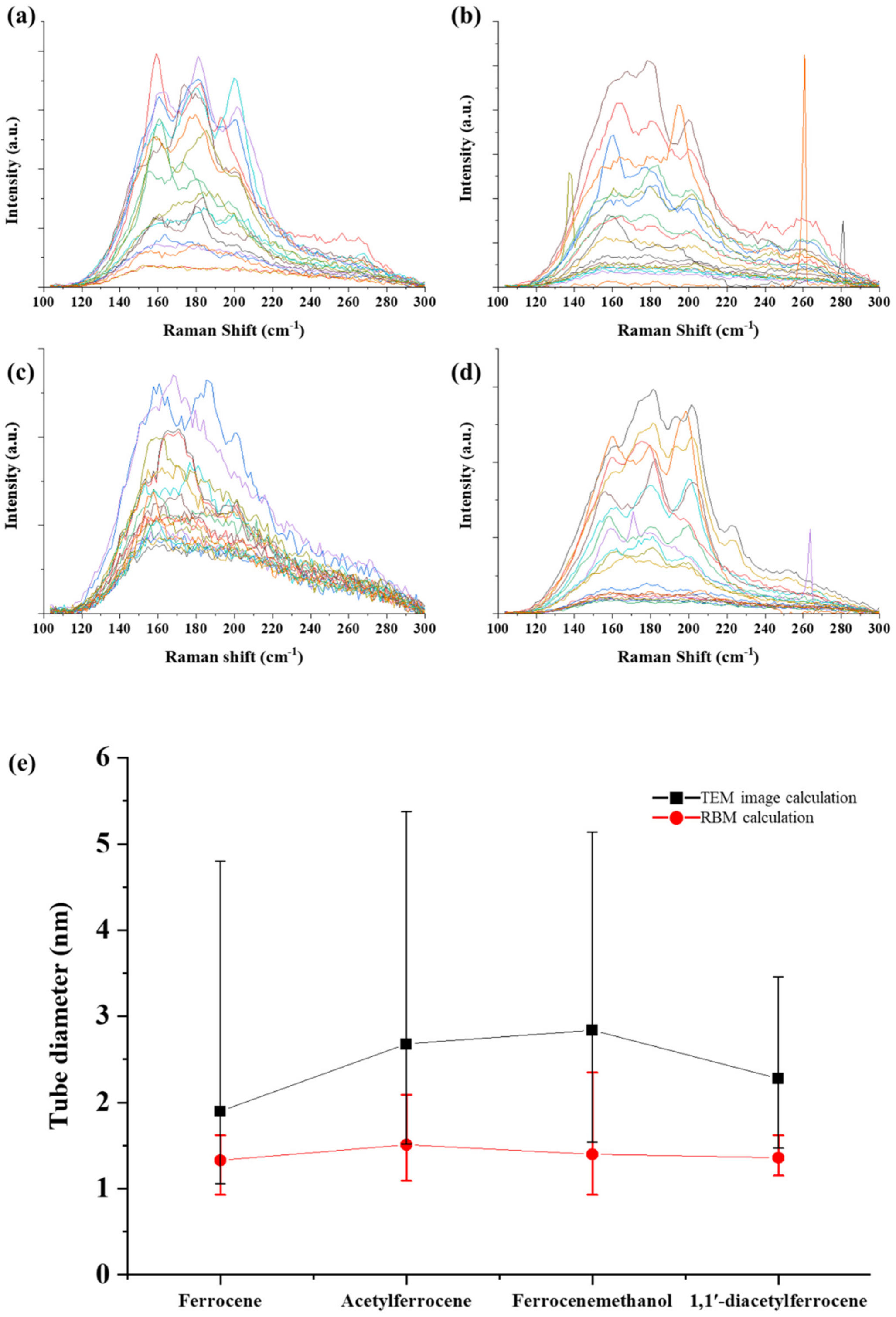

Figure S3. RBM mode of raman spectra ; (a) ferrocene, (b) acetylferrocene, (c) ferrocenemethanol, (d) 1,1′-diacetylferrocene, (e) calculated tube diameter according to RBM peak (—•—) and TEM image (—■—).

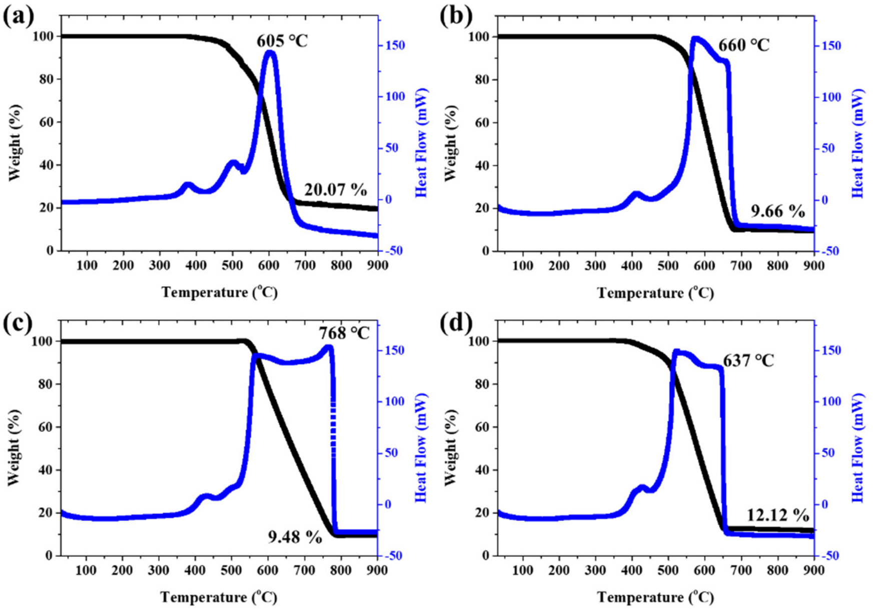

Figure S4. TG-DSC of the synthesized CNTs with catalyst precursors; (a) ferrocene, (b) acetylferrocene, (c) ferrocenemethanol, and (d) 1,1′-diacetylferrocene.

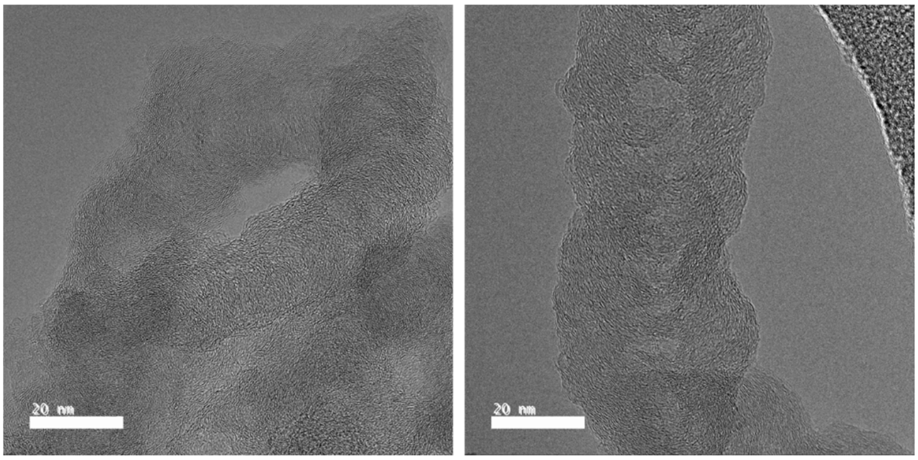

Figure S5. TEM images of carbon stacking morphology synthesized with 1,1′-diacetylferrocene.

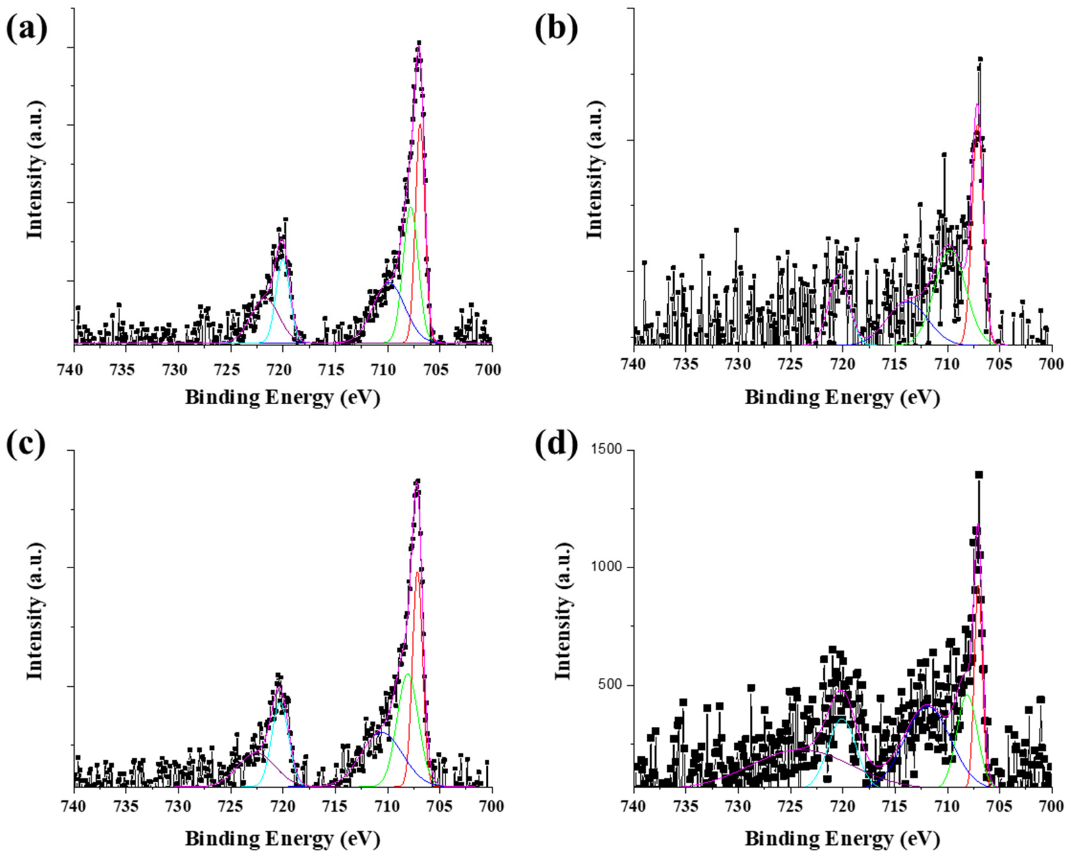

Figure S6. XPS spectra of Fe 2p of thermally decomposed catalyst precursors with sulfur; (a) ferrocene, (b) acetylferrocene, (c) ferrocenemethanol, and (d) 1,1′-diacetylferroce.

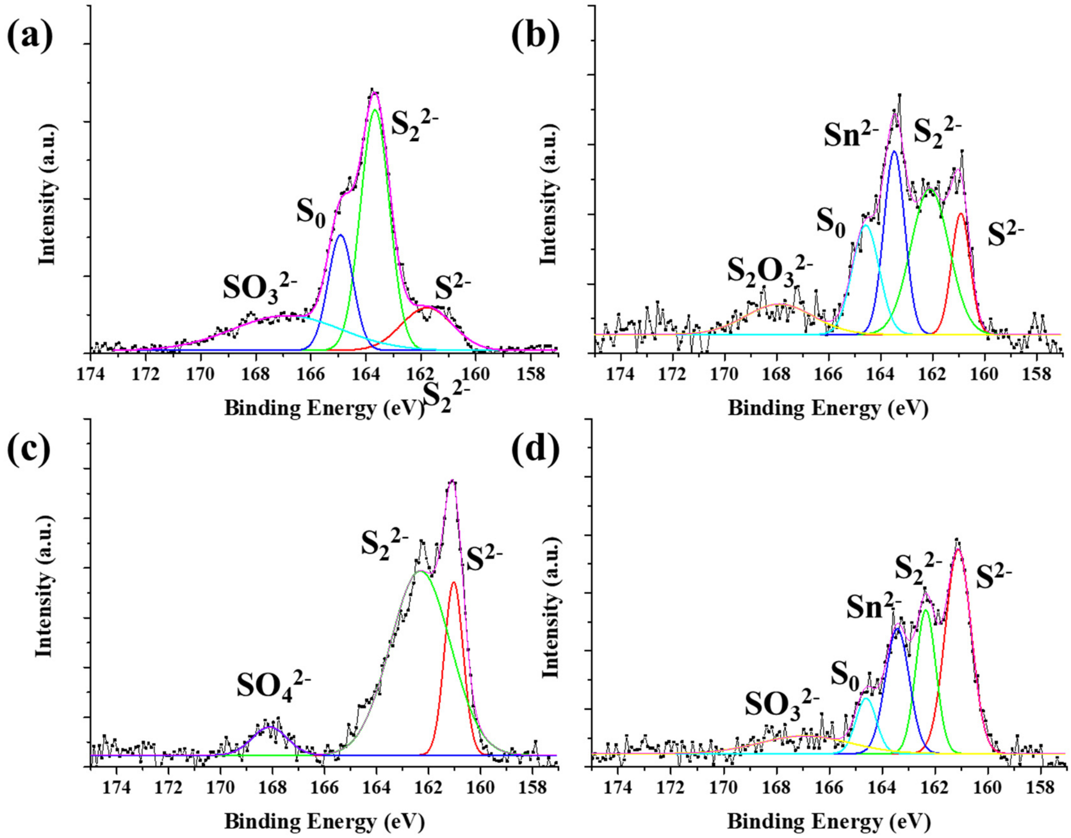

Figure S7. XPS spectra of S 2p of thermally decomposed catalyst precursors with sulfur; (a) ferrocene, (b) acetylferrocene, (c) ferrocenemethanol, and (d) 1,1′-diacetylferroce.
